# Retraction: Tumor Microenvironment in Oral Cancer Following Neoadjuvant Pembrolizumab: Preliminary Analysis of the Histopathologic Findings

**DOI:** 10.3389/froh.2022.893486

**Published:** 2022-03-22

**Authors:** Frontiers Editorial Office

The journal and Authors retract the 23 April 2021 article cited above for the following reason as provided by the Authors:

Following publication, the Authors were informed that they were not entitled to publish the raw data underlying the findings by the supplier of the analyzed drug.

Therefore, with the agreement of the Authors and the Chief Editor of the Oral Cancers specialty of Frontiers in Oral Health, the publication is being retracted.

